# Monitoring Cell Death in Regorafenib-Treated Experimental Colon Carcinomas Using Annexin-Based Optical Fluorescence Imaging Validated by Perfusion MRI

**DOI:** 10.1371/journal.pone.0138452

**Published:** 2015-09-22

**Authors:** Philipp M. Kazmierczak, Egon Burian, Ralf Eschbach, Heidrun Hirner-Eppeneder, Matthias Moser, Lukas Havla, Michel Eisenblätter, Maximilian F. Reiser, Konstantin Nikolaou, Clemens C. Cyran

**Affiliations:** 1 Institute for Clinical Radiology, Laboratory for Experimental Radiology, Ludwig-Maximilians-University Hospital Munich, München, Germany; 2 Institute for Clinical Radiology, Josef-Lissner-Laboratory for Biomedical Imaging, Ludwig-Maximilians-University Hospital Munich, München, Germany; 3 Department of Clinical Radiology, University Hospital Münster, Münster, Germany; 4 Comprehensive Cancer Imaging Centre, Division of Imaging Sciences & Biomedical Engineering, King’s College London, United Kingdom; 5 Department of Diagnostic and Interventional Radiology, University Hospital Tübingen, Tübingen, Germany; Case Western Reserve University, UNITED STATES

## Abstract

**Objective:**

To investigate annexin-based optical fluorescence imaging (OI) for monitoring regorafenib-induced early cell death in experimental colon carcinomas in rats, validated by perfusion MRI and multiparametric immunohistochemistry.

**Materials and Methods:**

Subcutaneous human colon carcinomas (HT-29) in athymic rats (n = 16) were imaged before and after a one-week therapy with regorafenib (n = 8) or placebo (n = 8) using annexin-based OI and perfusion MRI at 3 Tesla. Optical signal-to-noise ratio (SNR) and MRI tumor perfusion parameters (plasma flow PF, mL/100mL/min; plasma volume PV, %) were assessed. On day 7, tumors underwent immunohistochemical analysis for tumor cell apoptosis (TUNEL), proliferation (Ki-67), and microvascular density (CD31).

**Results:**

Apoptosis-targeted OI demonstrated a tumor-specific probe accumulation with a significant increase of tumor SNR under therapy (mean Δ +7.78±2.95, control: -0.80±2.48, p = 0.021). MRI detected a significant reduction of tumor perfusion in the therapy group (mean ΔPF -8.17±2.32 mL/100 mL/min, control -0.11±3.36 mL/100 mL/min, p = 0.036). Immunohistochemistry showed significantly more apoptosis (TUNEL; 11392±1486 vs. 2921±334, p = 0.001), significantly less proliferation (Ki-67; 1754±184 vs. 2883±323, p = 0.012), and significantly lower microvascular density (CD31; 107±10 vs. 182±22, p = 0.006) in the therapy group.

**Conclusions:**

Annexin-based OI allowed for the non-invasive monitoring of regorafenib-induced early cell death in experimental colon carcinomas, validated by perfusion MRI and multiparametric immunohistochemistry.

## Introduction

The multityrosine kinase inhibitor (MTKI) regorafenib is one of the most recent treatment options for patients with advanced colorectal cancer [1]. MTKI are a class of molecular agents known to exhibit significant effects on tumor physiology with only minor effects on tumor size, particularly in the early treatment phase [2]. Therefore, traditional, morphology-based response evaluation criteria such as the Response Evaluation Criteria In Solid Tumors (RECIST) are of limited value in MTKI-treated patients [3]. Recently, a decrease in tumor perfusion as assessed by DCE-CT or DCE-MRI has been proposed as a new imaging biomarker of therapy response for the anti-angiogenic effects of MTKI [4–6].

Beyond functional imaging, the investigation of molecular imaging techniques may convey a deeper understanding of tumor pathophysiology under therapy. Along with a significant suppression of tumor microperfusion, regorafenib exhibits significant pro-apoptotic effects on human colon carcinoma models [7–9]. Targeting the receptor tyrosine kinases vascular endothelial growth factor receptor (VEGFR) 1 to 3, angiopoietin-1 receptor (TIE2), and platelet-derived growth factor receptor (PDGFR) β, regorafenib inhibits tumor neovascularization and vessel stabilization [10]. Its pro-apoptotic effects in colorectal cancer are mediated by induction of p53-upregulated modulator of apoptosis (PUMA), leading to dysfunction of mitochondria and caspase activation [7]. In addition, regorafenib has been shown to enhance SH2-domain-containing phosphatase 1 (SHP-1) activity, leading to an inhibition of STAT3 and therefore increasing apoptosis [11]. Thus, apoptosis is a promising target for the non-invasive characterization of the tumor microenviroment of experimental colon carcinomas under regorafenib therapy in vivo. Near-infrared range (NIR) optical fluorescence imaging (OI) with targeted probes is a preclinical molecular imaging modality allowing for the non-invasive visualization of tumor cell apoptosis, generating real-time information on tumor pathophysiology which to date can only be obtained by histopathological analysis *ex vivo* [12]. Hence, adding apoptosis-targeted OI to a multiparametric MRI protocol would build a novel molecular hybrid imaging concept for the real-time and non-invasive assessment of early therapy effects beyond the morphological and functional level.

We hypothesized that a one-week regorafenib monotherapy would exhibit significant pro-apoptotic effects on experimental colon carcinoma xenografts in rats as assessed by OI with an apoptosis-targeted probe, validated by DCE-MRI and multiparametric immunohistochemistry. The aim of the study was to investigate if apoptosis-targeted OI can provide information on early therapy response of experimental colon carcinomas to MTKI therapy in vivo.

## Materials and Methods

The trial received approval by the Government (Regierung von Oberbayern, Germany) Committee for Animal Research (Gz.: 55.2-1-54-2532-33-10). It was conducted in accordance with the guidelines of the National Institute of Health for the Care and Use of Laboratory Animals. Animals were kept in individually-ventilated cage systems (n = 4 per cage) at a constant temperature of 26°C, a relative humidity of 65%, and 18 room air changes per hour. The light-dark-cycle was 12 hours. Animals were nourished ad libitum with water and dedicated small animal nutrition. For environmental enrichment purposes, rat nest boxes were provided. Animals were euthanized under inhalation anesthesia (5.0% isoflurane in pure oxygen) by intracardial injection of a saturated potassium chloride solution. Every effort was made in order to avoid animal suffering.

### Animal model and examination protocol

Athymic nude rats (n = 16, 7–8 weeks old, Harlan Laboratories Inc., Indianapolis, IN) were subcutaneously injected with 3 × 10^6^ cells of the human colon carcinoma cell line HT-29 (ATCC HTB-38) which were suspended in 0.5 mL of equal parts of Matrigel™ (BD Biosciences, San Jose, CA) and phosphate buffered saline (PBS, pH 7.4). After reaching a tumor diameter of ≥ 1.0 cm, animals were randomly assigned to either the therapy (n = 8) or the control group (n = 8). Before initiation of treatment, both control and therapy group were scanned by DCE-MRI and OI (day 0, baseline examination). MRI contrast agent and OI probe were administered via intravenous tail vein catheter (butterfly catheter, 25-gauge, B. Braun AG, Melsungen, Germany). The examinations were performed under inhalation anaesthesia (2.5% to 5.0% isoflurane in pure oxygen). The OI probe was injected 2 h prior to the OI scan as recommended by the manufacturer and as confirmed by preliminary kinetic studies. In the meantime, animals were transferred to the MRI suite where DCE-MRI measurements were performed. Immediately after the MRI scan, the animals were transferred to the laboratory where the OI scan was performed. Detailed imaging protocols for DCE-MRI and OI are provided below. From day 1 to 6, animals were treated with either regorafenib (therapy group) or placebo (control group). On day 7, the multimodality DCE-MRI/OI imaging protocol was repeated as described above (follow-up examination). After euthanization of the animals on day 7 and subsequent tumor explantation, the tumor material was fixed in formalin for immunohistochemical analysis.

### Treatment

Regorafenib was solved in polypropylene glycol/PEG400/Poloxamer 188 (42.5/42.5/15 + 20% Aqua). The therapy group was treated with a daily dose of 10 mg regorafenib per kg body weight via gastric gavage while the control group received a volume-equivalent placebo.

### OI protocol

OI was performed on a fully automated preclinical small animal optical imaging system (In-Vivo FX PRO, Bruker Corp., Billerica, MA) using a charge-coupled device (CCD) camera (2048 x 2048 pixels). The commercially available apoptosis-targeted probe Annexin Vivo 750 (Perkin Elmer, Waltham, MA) was injected manually via the lateral tail vein at a dose of 16 nmol, according to the manufacturer’s recommendation. Preliminary kinetic studies demonstrated that peak intra-tumor annexin accumulation was 2 h after intravenous injection, confirming the manufacturer’s recommendation. Annexin Vivo 750 is built of a NIR fluorochrome conjugated to Annexin A5, a cellular protein selectively binding to phosphatidylserine which is expressed on the phospholipid bilayers of cell membranes during the early stages of apoptosis [13]. According to the manufacturer, tissue half-life time of Annexin Vivo 750 is 14 h. Naïve scans documented full tumor clearance of the probe after a follow-up period of one week. Residual fur of the athymic nude rats was removed in a dedicated depilatory procedure in order to avoid scattering and absorption artifacts [14]. Animals were scanned in the lateral position with the subcutaneous tumor placed directly on the examination plane. In pilot fluorescence studies, an optimal SNR was delivered at an imaging time of 30 s. The comprehensive multi-step optical imaging protocol consisted of an x-ray (step 1, exposure time 1 s), a white-light (step 2, excitation filter 410 nm, no emission filter, field-of-view 200.0 mm, focal plane 11.0 mm, f/2.5, resolution 260 ppi x 260 ppi, exposure time 1 s), and a fluorescence scan (step 3, excitation filter 730 nm, emission filter 790 nm, field-of-view 200.0 mm, focal plane 11.0 mm, 2x x-binning, 2x y-binning, resolution 130 ppi x 130 ppi, exposure time 30 s), with a total scan duration of 7 min. A naïve scan was performed for the differentiation of signal from background noise.

### OI data and image analysis

Using dedicated molecular imaging post-processing software (Bruker Molecular Imaging Software, Bruker Corp., Billerica, MA), a region of interest (ROI) was placed over the tumor (target tissue), the thigh muscle (non-target tissue), and the background beside the mouse. OI signal-to-noise ratio (SNR) was calculated using a formula published previously [15], where SI is the mean signal intensity normalized for tumor area and SD the standard deviation of the background noise within a defined ROI: SNR = (SI_tumor_−SI_non-target tissue_)/SD_background_. Signal intensity was expressed as arbitrary units [a.u.].

### DCE-MRI

MRI examinations were performed on a clinical 3 Tesla scanner (Magnetom Verio, Siemens Healthcare, Erlangen, Germany). Animals were examined in supine position using a four-channel small-animal coil (Rapid MR International, Columbus, OH). The clinical low molecular contrast agent Gadobutrol (Gadovist®, Bayer HealthCare, Leverkusen, Germany) was administered intravenously by a dedicated automated small-animal injection pump (Harvard Apparatus PHD 2000, Instech Lab. Inc., Plymouth Meeting, PA) at a dose of 0.1 mmol/kg body weight with an injection speed of 7.2 mL/min and a subsequent 0.5 mL saline chaser. A 3D view-sharing sequence (Time-Resolved Angiography With Stochastic Trajectories, TWIST, Siemens Healthcare, Erlangen, Germany; temporal resolution 1.3 s) was performed for assessment of tumor contrast kinetics. After acquisition of 10 non-enhanced frames within 13 s, 290 additional contrast-enhanced frames were obtained. The protocol rendered 300 high-resolution 3D data sets. Sequence parameter were as follows: spatial resolution 0.39 mm x 0.39 mm x 3.0 mm, repetition time/echo time 6.34/2.11 ms, 128 x 128 matrix, field-of-view 50 mm x 50 mm, flip angle 40°, total acquisition time 6:30 min).

### MRI Data Processing–kinetic analysis

Data post-processing was performed on a PMI (Platform for Research in Medical Imaging) workstation, with software written in-house in IDL 6.4 (ITT Visual Information Solutions, Boulder, CO). To generate an arterial input function (AIF), a blood ROI was placed over the inferior vena cava at the level of the liver. The tumor ROI was placed over the tumor periphery, defined as the outer 2 mm of the tumor in which high interstitial pressure and necrosis are less likely to occur [4]. Signal intensity was plotted versus time for the approximation of probe concentrations in relation to their relative enhancement. Fitting the data to a two-compartment uptake model allowed for the calculation of plasma flow (PF, mL/100 mL/min, surrogate of tumor perfusion) and plasma volume (PV, %, surrogate of tumor vascularity). For assessments of tumor sizes pre and post therapy, a voxel-based volumetry was performed.

### Immunohistochemistry

Tissue samples fixed in formaldehyde and embedded in paraffin were heated to 60°C and washed in xylene substitute (Neo-Clear, Merck KgaA, Darmstadt, Germany) for de-waxing, then re-hydrated in ethanol at different concentrations (100%, 96%, 90%, 70%) and distilled water. Subsequent to tissue demasking by microwave irradiation at 600 W in a 0.1 M citrate buffer solution (pH 6.0), dedicated staining was performed with regard to microvascular density (CD31), tumor cell proliferation (Ki-67), and tumor cell apoptosis (Terminal Deoxynucleotidyl Transferase-mediated Nick End Labeling, TUNEL). Results were expressed as the average number of positively-stained structures in 10 random fields at a magnification of 200x.

#### CD31

Tissue demasking was performed in a 0.1 M citrate buffer solution with microwave irradiation at 600 W. Following overnight incubation with the primary antibody (ab28364, 1:50), a commercially available kit (EnVision+ System HRP (AEC), Dako, Germany) was applied for further work-up.

#### Ki-67

Tissue demasking was performed in a 0.1 M citrate buffer solution with microwave irradiation at 600 W. Tumor cell proliferation was quantified by a monoclonal rabbit anti-rat antibody specific for Ki-67. For permeabilization purposes, slides were immersed in 0.25% Triton X-100 –Tris-Cl solution. A commercial multi-step kit (Dako EnVision+ HRP (DAB), Dako, Germany) was employed for secondary antibody application to the primary antibody (ab16667, 1:100).

#### TUNEL

Tissue samples were prepared using a dedicated apoptosis detection kit (in situ Cell Death Detection Kit, Roche Diagnostics, Indianapolis, IN) and subsequently analyzed by fluorescence microscopy (standard fluorescent filter, 520±20 nm).

### Statistical Analysis

Outcome parameters were SNR (OI), PF (mL/100 mL/min, DCE-MRI), PV (%, DCE-MRI), and the positively-stained structures per high-power field (immunohistochemistry), expressed as means with standard errors (SE). The parameters were also expressed as differences (Δ) of post minus pre therapy values (ΔSNR, ΔPF, ΔPV). For intra- and inter-group comparisons, a Wilcoxon signed-rank test was applied. Correlations between imaging and immunohistochemical parameters were assessed by Spearman’s rho. Statistical significance was assumed for p-values <0.05.

## Results

Measurements were successfully completed in 16 animals (therapy group, n = 8; control group, n = 8).

### Optical fluorescence imaging

**Fig 1:**
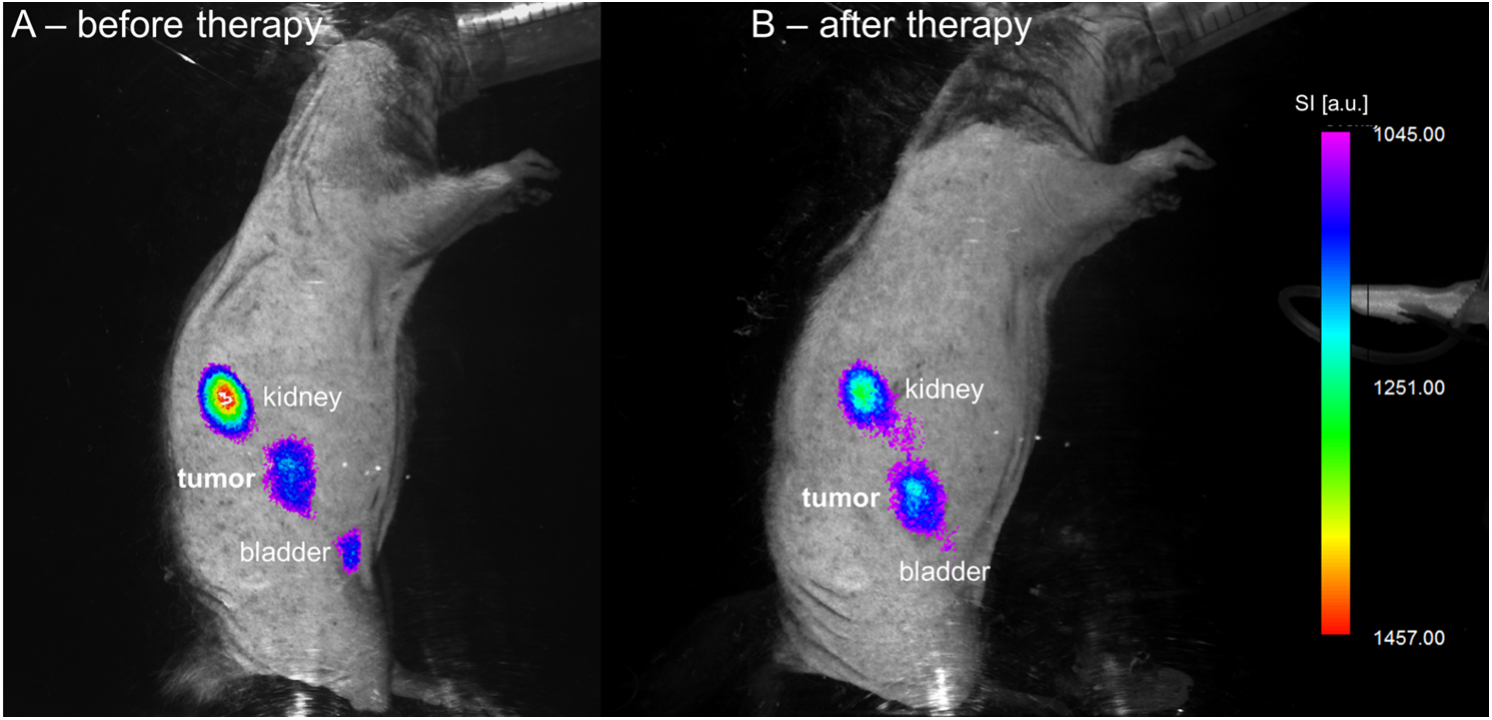
Cell death-related OI signal before and after treatment. Fusion of color-coded fluorescence and anatomic white-light image of one animal before (A) and after (B) a one-week regorafenib monotherapy. Note the significant increase in cell death-related signal under therapy, predominantly in the center of the tumor. High signal intensity is also observed in the kidneys and the urinary bladder due to renal probe elimination. OI signal intensity (SI) is displayed in arbitrary units (a.u.).

**Fig 2:**
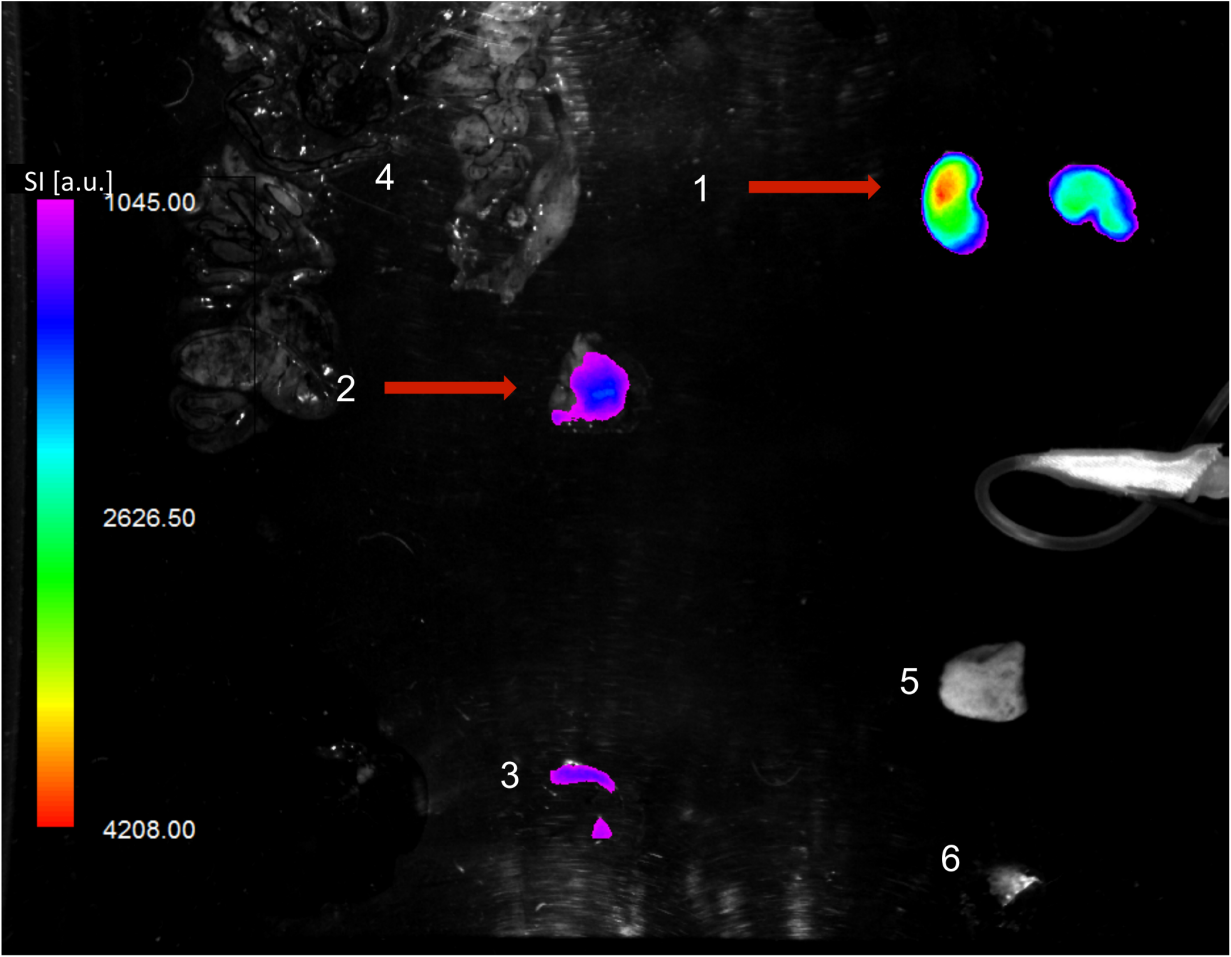
*Ex vivo* OI analysis of explanted main organs. Fusion of color-coded fluorescence and anatomic white-light image. In accordance with the in vivo results, highest OI signal is observed in the kidneys (1) and the tumor (2) (red arrows). As a blood pool organ, the spleen (3) demonstrates relatively high probe accumulation. Only subtle OI signal is detected in the gut (4), the skin (5), and the thigh muscle (6). OI signal intensity (SI) is displayed in arbitrary units (a.u.).

**Fig 3:**
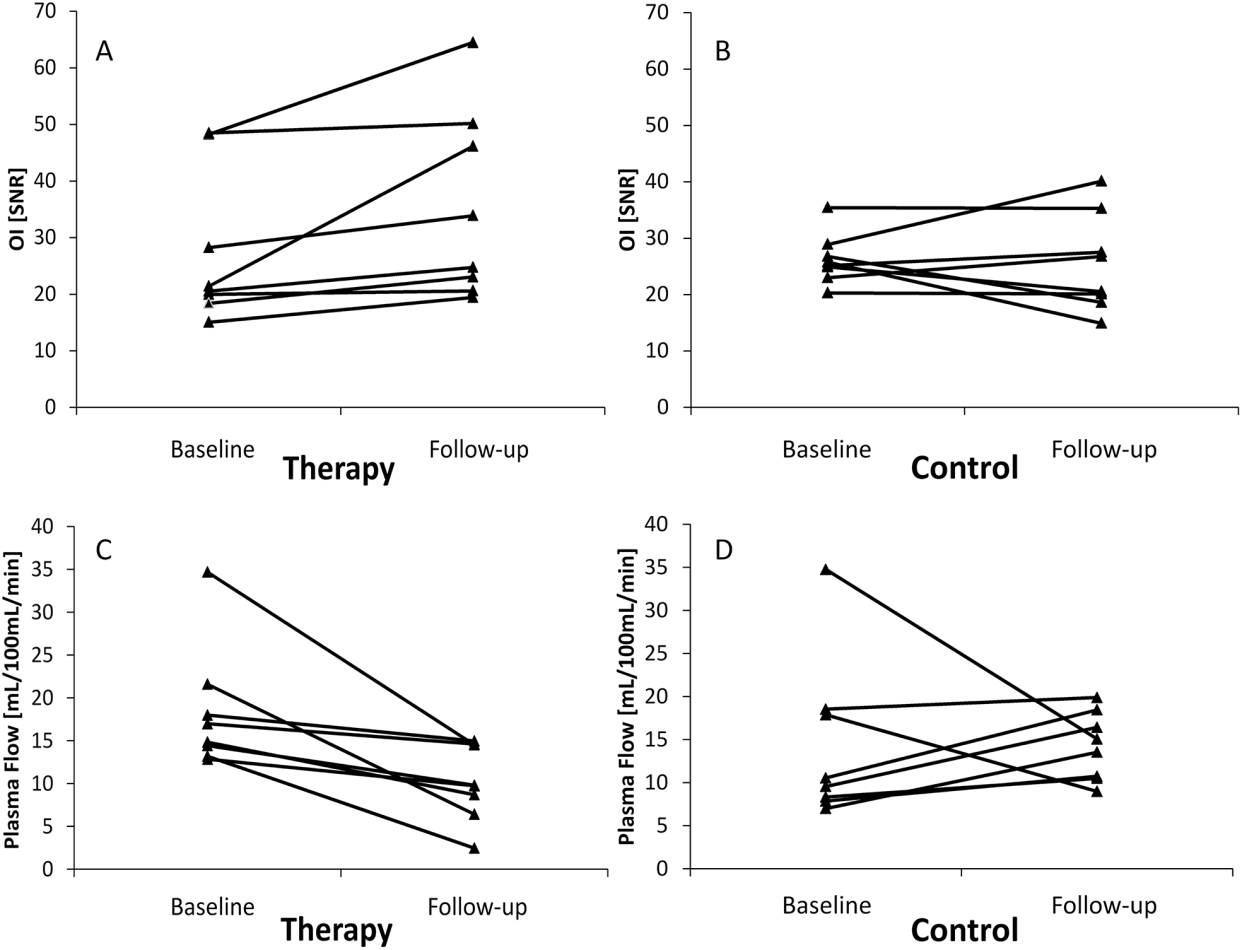
Development of individual OI and MR perfusion values. Line graphs depicting development of individual optical fluorescence imaging (OI, SNR) and plasma flow (PF, mL/100 mL/min) values between baseline and follow-up. Note the unidirectional increase of OI SNR (A) and the unidirectional suppression of PF (C) under therapy. On the contrary, omnidirectional development of individual OI SNR (B) and PF (D) was observed in the control group.

There was no significant difference in pre-treatment values between therapy and control group (mean pre-treatment SNR: therapy group 27.6 ± 4.7, control group 26.3 ± 1.6, p = 0.401). Regorafenib therapy lead to a significant, unidirectional increase of absolute SNR between baseline and follow-up (mean SNR from 27.6 ± 4.7 to 35.3 ± 5.9; p = 0.012). On the contrary, absolute SNR values developed heterogeneously and non-significantly in the control group (mean SNR from 26.3 ± 1.6 to 25.5 ± 3.1, p = 0.674). A significant difference in ΔSNR between therapy and control group was observed (mean ΔSNR: therapy group +7.8 ± 2.9, control group -0.8 ± 2.5, p = 0.021). Fig 1 shows the increase of annexin-related OI signal under regorafenib therapy in one animal (Fig 1). High signal intensity was also observed in the kidney and the urinary bladder due to renal probe elimination. Accordingly, *ex vivo* OI analysis of the main organs revealed highest probe accumulation in the tumor and the kidneys (Fig 2). See Tables 1 and 2 for individual OI values pre and post treatment, also displayed as line graphs in Fig 3.

**Table 1:** Individual OI and MR perfusion values at baseline and follow-up (therapy group). Note the significant increase of apoptosis-targeted OI signal and the significant plasma flow (PF) reduction in the therapy group (p < 0.05).

Animal no.	^a^OI	^b^OI *	^a^PF	^b^PF *	^a^PV	^b^PV
1	48.3	64.5	34.7	14.5	10.1	8.7
2	48.5	50.2	13.2	2.5	5.4	4.6
3	21.4	46.2	12.9	9.8	19.9	10.6
4	20.6	24.8	18.0	15.0	7.3	3.2
5	20.0	20.6	14.8	8.7	19.6	7.8
6	18.4	23.1	14.4	9.8	22.6	11.7
7	15.1	19.5	21.6	6.4	6.9	9.7
8	28.3	33.9	17.0	14.6	14.0	13.9
**mean**	27.6	35.3	18.3	10.2	13.2	8.8
**SE**	4.8	5.9	2.6	1.6	2.4	1.3

^a^baseline

^b^follow-up; SE = standard error

*p < 0.05 (follow-up vs. baseline)

**Table 2:** Individual OI and MR perfusion values at baseline and follow-up (control group). Note the omnidirectional development of individual apoptosis-targeted OI signal and perfusion parameters.

Animal no.	^a^OI	^b^OI	^a^PF	^b^PF	^a^PV	^b^PV
9	35.4	35.3	34.8	15.1	8.0	15.8
10	23.0	26.8	7.0	13.6	17.4	7.9
11	28.9	40.1	17.9	9.0	7.6	4.7
12	25.2	27.5	9.6	16.4	8.2	20.4
13	24.9	20.6	7.9	10.7	9.5	14.0
14	20.3	20.1	8.3	10.5	9.0	8.7
15	25.8	14.9	10.6	18.5	23.8	35.6
16	26.8	18.6	18.5	19.9	10.7	8.8
**mean**	26.3	25.5	14.3	14.2	11.8	14.5
**SE**	1.6	3.0	3.3	1.4	2.1	3.5

^a^baseline

^b^follow-up; SE = standard error

*p < 0.05 (follow-up vs. baseline)

### Perfusion MRI

**Fig 4:**
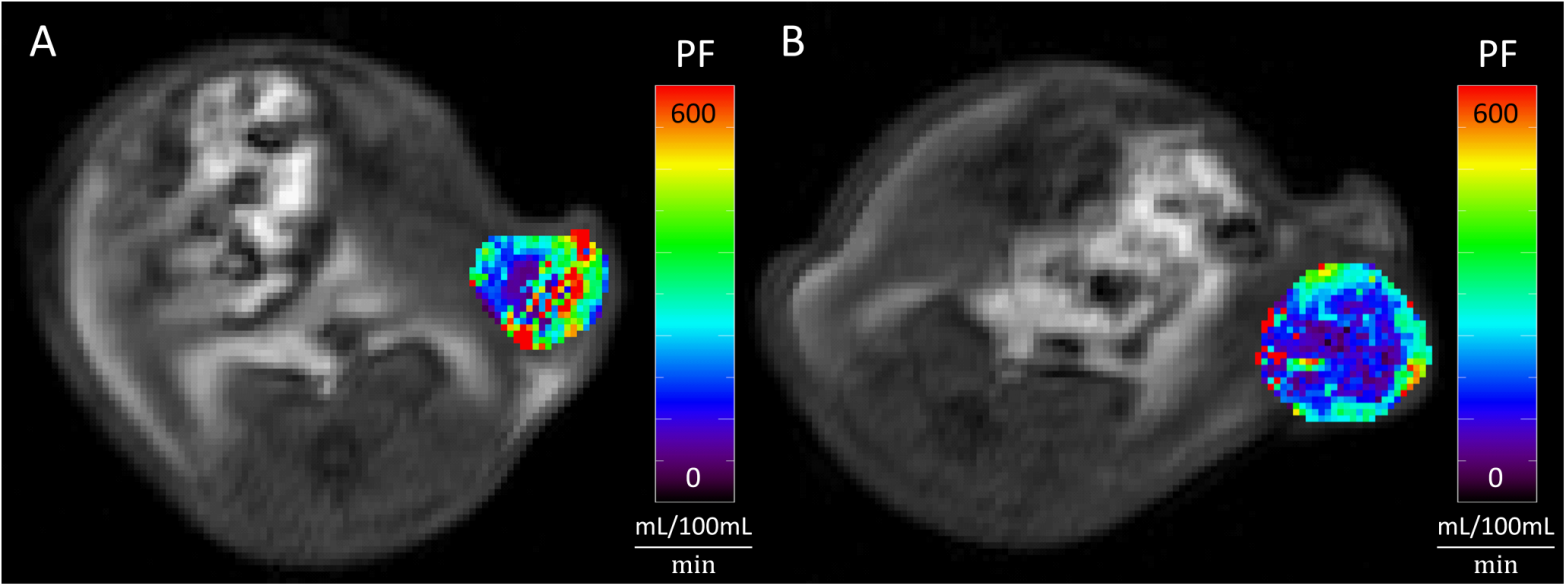
Decrease of tumor perfusion following anti-angiogenic therapy. Tumor perfusion (Plasma Flow, PF) before (A) and after (B) a one-week regorafenib treatment of a subcutaneous colon carcinoma xenografts over the left abdominal flank. Note the significantly reduced plasma flow after therapy (B vs. A).

No significant difference in pre-treatment plasma flow values between therapy and control group was observed (mean pre-treatment PF: therapy group 18.3 ± 2.5 mL/100 mL/min, control group 14.3 ± 3.3 mL/100 mL/min, p = 0.172). Under therapy, a significant, unidirectional suppression of PF was detected between day 0 and day 7 (mean PF: baseline 18.3 ± 2.5 mL/100 mL/min, follow-up 10.2 ± 1.6 mL/100 mL/min, p = 0.012). No significant change in PF between baseline and follow-up was observed in the control group (mean PF: baseline 14.3 ± 3.3 mL/100 mL/min, follow-up 14.2 ± 1.4 mL/100 mL/min, p = 0.674). ΔPF differed significantly between therapy and control group, showing a significant suppression of tumor perfusion under therapy (mean ΔPF: therapy group –8.2 ± 2.3 mL/100 mL/min, control group –0.1 ± 3.4 mL/100 mL/min, p = 0.036). No statistically significant effects were observed on PV. Fig 4 shows an example of tumor PF suppression in one animal from the therapy group (Fig 4). Individual values of PF in the therapy and the control group are displayed in Tables 1 and 2 and as line graphs in Fig 3.

### Immunohistochemistry

**Fig 5:**
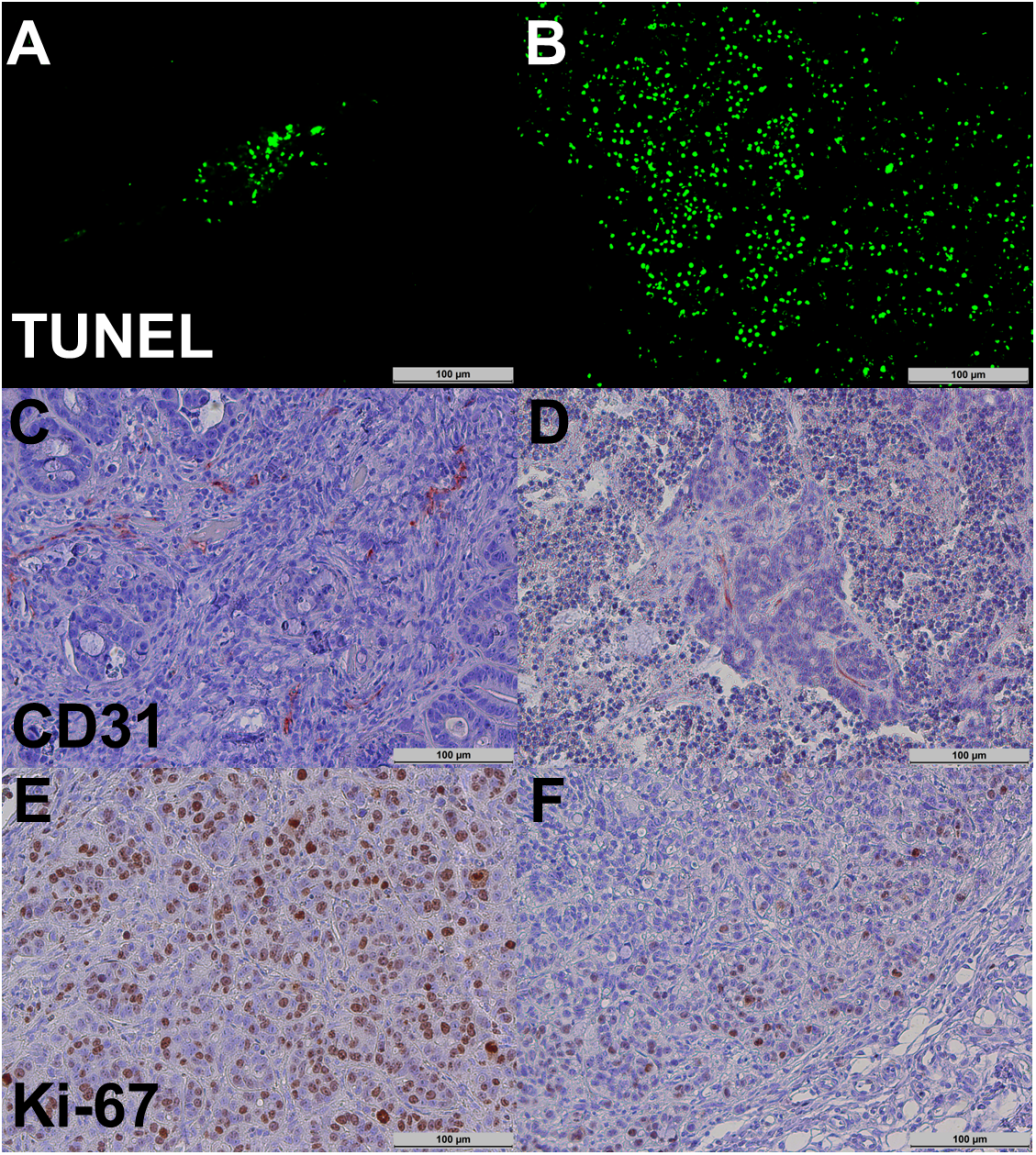
Representative immunohistochemical stainings. Tumor cell apoptosis (TUNEL, A and B), tumor microvascular density (CD31, C and D), and tumor cell proliferation (Ki-67, E and F) in the control (left column; A, C, and E) and the therapy group (right column; B, D, and F). Note the significantly higher apoptosis rate (B vs. A) as well as the significantly lower microvascular density (D vs. C) and proliferation (F vs. E) in the therapy group.

Immunohistochemical analysis revealed a significantly higher rate of apoptosis in the regorafenib-treated group (mean number of cells stained positive for TUNEL: therapy group 11392 ± 1486, control group 2921 ± 334, p = 0.001). Likewise, a significant suppression of tumor cell proliferation (mean number of cells stained positive for Ki-67: therapy group 1754 ± 184, control group 2883 ± 323, p = 0.012) and tumor vascularity (mean number of cells stained positive for CD31: therapy group 107 ± 10, control group 182 ± 22, p = 0.006) was observed. Individual values for the immunohistochemical parameters are provided in Table 3. Fig 5 displays representative immunohistochemical stainings of one tumor from the therapy group.

**Table 3:** Individual immunohistochemical values in the control (animals no. 1 to 8) and the therapy group (animals no. 9 to 16). Note the significantly lower microvascular density (CD31) and proliferation (Ki-67) as well as the significantly higher apoptosis rate in the therapy group (p < 0.05).

Animal no.	CD31	TUNEL	Ki-67
1	203	2020	2699
2	162	2902	2488
3	282	2551	3821
4	124	2269	1975
5	174	2206	3204
6	136	4923	1566
7	114	3514	4331
8	261	2982	2982
**Mean**	182	2921	2883
**SE**	22	334	323
9	144	6239	1299
10	79	7614	1336
11	101	8010	1639
12	160	10791	2245
13	98	18505	2536
14	85	11983	1458
15	100	15762	2293
16	93	12301	1233
**Mean**	107*	11392*	1754*
**SE**	10	1486	184

SE = standard error

*p < 0.05 (control vs. therapy group)

### Tumor sizes in treatment and control group

We did not observe any significant differences in initial tumor size between the two groups (therapy group 881±361cm^3^, control group 669±79 cm^3^; p = 0.6). No correlation between changes in tumor volume pre/post therapy and OI SNR were observed (Spearman’s rho -0.115, p = 0.672).

### Correlations between non-invasive imaging biomarkers and immunohistochemistry

We observed a significant inverse correlation between OI_diff_ and proliferation as assessed by Ki-67 (Spearman’s rho -0.538, p = 0.031). OI_diff_ also showed a moderate but not significant positive correlation with apoptosis assessed by TUNEL (Spearman’s rho 0.385, p = 0.141). An inverse correlation of OI_diff_ and PF_diff_ was observed which proved borderline-significant (Spearman’s rho -0.474, p = 0.064). No other significant correlations were observed.

## Discussion

In this experimental study, OI with fluorescence-labeled Annexin A5 was investigated in a human colon carcinoma model in rats. Annexin-based OI allowed for the non-invasive visualization and quantification of early cell death induced by a regorafenib monotherapy in vivo, as validated by DCE-MRI and multiparametric immunohistochemistry.

### Optical Fluorescence Imaging

Annexin-based OI generated non-invasive surrogates of regorafenib-induced tumor cell death in vivo as validated by dedicated immunohistochemistry (TUNEL). These findings are in line with trials investigating annexin-based OI probes for the monitoring of cytotoxic and epidermal growth factor receptor (EGFR)-targeted therapies in vivo [16,17]. Additionally, they are in accordance with recent studies demonstrating that radiolabeled Annexin A5 allows for the in vivo monitoring of cell death in experimental lung cancer treated by cytotoxic therapy as well as in animal models of myocardial infarction [18,19]. Our results provide evidence for the applicability of fluorescence-labeled Annexin A5 for the monitoring of anti-angiogenic therapy regimes in vivo.

Contrarily, in a recent preclinical study investigating early therapy effects of MTKI sunitinib in a human squamous cell carcinoma model in mice, annexin-based optical and gamma imaging failed to detect early pro-apoptotic therapy effects [20]. The authors concluded that annexin-based imaging was not suitable for monitoring effects of anti-angiogenic therapies leading to a substantial breakdown of tumor vasculature, as consecutively the probe would not be delivered to the tumor microenvironment. According to this conclusion, one would expect a reduced annexin-related signal after anti-angiogenic treatment. However, we found a significant increase of annexin-related signal in the therapy group, making unspecific accumulation of the probe unlikely. Another possible explanation for the divergent findings in our study may be the longer follow-up interval of seven vs. four days, which may allow for a partial reformation and normalization of tumor microvasculature. Additionally, applicability of NIR agents as well as vascular responsiveness to anti-angiogenic treatment may vary between different tumor models. For instance, NIR blood pool agents detected early anti-angiogenic effects of sunitinib in experimental squamous cell carcinomas, but failed to show treatment-induced vascular changes in experimental breast carcinomas, although significant anti-angiogenic effects were proven by DCE-MRI, CEUS, and immunohistochemistry [20,21].

Our results suggest that annexin-based is able to generate non-invasive and real-time molecular data on tumor cell death in vivo. However, several issues remain to be addressed before OI can be translated into clinical routine.

#### Clinical limitations of OI

One major limitation of OI is the low penetration depth of only several centimeters [22], restricting the use to superficially-located structures. Several techniques for the direct on-surface application of OI have been established, including transcatheter, cystoscopic, endoscopic, and laparoscopic approaches [22–25]. Moreover, intraoperative OI has been applied successfully for the differentiation of healthy and tumor-invaded brain tissue [26–28]. Likewise, as regorafenib has been granted approval for the treatment of advanced colorectal carcinoma (i.e., hepatic metastazisation), one would have to find a similar way to successfully apply OI in the upper abdomen [29], where overlaying bones (i.e., ribs) and respiratory motion artifacts during the relatively long acquisition time may additionally compromise the clinical applicability.

#### Limitations of annexin-based imaging

Annexin A5 is known for its unspecific binding to abdominal organs and its slow tissue clearance, resulting in high background noise [13,30,31]. *Ex vivo* analysis confirmed this limitation (Fig 2). However, this may be excluded by unenhanced scans prior to probe administration, as performed in the present study. Despite, Annexin A5 is not immunogenic and has already been used clinically in radiolabeled PET tracers, factors facilitating clinical translation of annexin-based OI probes [13,31].

Depending on the phagocytic activity of auxiliary scavenger cells, apoptosis may be succeeded by autolysis, i.e., secondary necrosis [32]. During secondary necrosis with consecutive loss of cell membrane integrity, primarily intramembraneous phosphatidylserine is externalized and may thus be bound by Annexin A5. Consequently, annexin-based imaging is limited in its ability to distinguish between apoptosis and necrosis; however, it can still be considered an accurate measure of cell death. Several immunohistochemical techniques have been proposed for the differentiation of apoptosis from secondary necrosis *ex vivo*, e.g., using propidium iodide and necrostatin [33,34]. The applicability and additional value of these agents as part of a multimodal, fluorescence-based imaging protocol for the assessment of therapy-induced cell death in vivo should be investigated in future studies.

### Perfusion MRI

DCE-MRI allowed for the timely and non-invasive assessment of regorafenib-induced suppression of tumor perfusion in the therapy group, supporting recent data stating similar effects in an orthotopic colon carcinoma model in mice [9].

### Immunohistochemistry

Immunohistochemical results are in accordance with recent preclinical trials investigating regorafenib effects on a molecular level, demonstrating a significant reduction of microvascular density and proliferation under therapy [7–9,35]. Most strikingly, there is wide evidence that regorafenib acts as potent inductor of apoptosis, indicated by dedicated stainings for tumor cell apoptosis (TUNEL), analogously to the present study [7–9].

### Limitations

The results of our study are limited in several aspects. First, an orthotopic tumor model may represent the pathophysiology of human cancer better and may potentially allow for the additional in vivo assessment of metastasis formation. Second, a broader range of immunohistochemical markers may supply additional data with regard to the molecular effects of regorafenib on the tumor microenvironment. Third, only one combination of a tumor model and a molecular cancer therapy has been investigated, and imaging has only been performed after a follow-up time of seven days.

### Conclusions

In conclusion, annexin-based OI allowed for the early and non-invasive monitoring of a regorafenib monotherapy in experimental colon carcinomas in rats, as validated by perfusion MRI and multiparametric immunohistochemistry. Thus, annexin-based OI may be applied for the non-invasive characterization of early tumor cell death under therapy in vivo. Combining morphology-based, functional, and molecular imaging techniques, future multimodality imaging protocols may facilitate patient assignment to targeted cancer therapies by the timely and non-invasive differentiation of responders and non-responders.

## References

[1] Grothey A, Van Cutsem E, Sobrero A, Siena S, Falcone A, Ychou M, et al Regorafenib monotherapy for previously treated metastatic colorectal cancer (CORRECT): an international, multicentre, randomised, placebo-controlled, phase 3 trial. Lancet. 2013;381: 303–312. 10.1016/S0140-6736(12)61900-X 23177514

[2] Ratain MJ, Eckhardt SG. Phase II studies of modern drugs directed against new targets: if you are fazed, too, then resist RECIST. J Clin Oncol. 2004;22: 4442–4445. 15483011 10.1200/JCO.2004.07.960

[3] Desar IM, van Herpen CM, van Laarhoven HW, Barentsz JO, Oyen WJ, van der Graaf WT. Beyond RECIST: molecular and functional imaging techniques for evaluation of response to targeted therapy. Cancer Treat Rev. 2009;35: 309–321. 10.1016/j.ctrv.2008.12.001 19136215

[4] Cyran CC, von Einem JC, Paprottka PM, Schwarz B, Ingrisch M, Dietrich O, et al Dynamic contrast-enhanced computed tomography imaging biomarkers correlated with immunohistochemistry for monitoring the effects of sorafenib on experimental prostate carcinomas. Invest Radiol. 2012;47: 49–57. 10.1097/RLI.0b013e3182300fe4 21934514

[5] Cyran CC, Paprottka PM, Schwarz B, Sourbron S, Ingrisch M, von Einem J, et al Perfusion MRI for monitoring the effect of sorafenib on experimental prostate carcinoma: a validation study. AJR Am J Roentgenol. 2012;198: 384–391. 10.2214/AJR.11.6951 22268182

[6] Messiou C, Orton M, Ang JE, Collins DJ, Morgan VA, Mears D, et al Advanced solid tumors treated with cediranib: comparison of dynamic contrast-enhanced MR imaging and CT as markers of vascular activity. Radiology. 2012;265: 426–436. 10.1148/radiol.12112565 22891356

[7] Chen D, Wei L, Yu J, Zhang L. Regorafenib inhibits colorectal tumor growth through PUMA-mediated apoptosis. Clin Cancer Res. 2014;20: 3472–3484. 10.1158/1078-0432.CCR-13-2944 24763611 PMC4079733

[8] Cyran CC, Kazmierczak PM, Hirner H, Moser M, Ingrisch M, Havla L, et al Regorafenib effects on human colon carcinoma xenografts monitored by dynamic contrast-enhanced computed tomography with immunohistochemical validation. PLoS One. 2013;8: e76009 10.1371/journal.pone.0076009 24098755 PMC3786893

[9] Abou-Elkacem L, Arns S, Brix G, Gremse F, Zopf D, Kiessling F, et al Regorafenib inhibits growth, angiogenesis, and metastasis in a highly aggressive, orthotopic colon cancer model. Mol Cancer Ther. 2013;12: 1322–1331. 10.1158/1535-7163.MCT-12-1162 23619301

[10] Wilhelm SM, Dumas J, Adnane L, Lynch M, Carter CA, Schutz G, et al Regorafenib (BAY 73–4506): a new oral multikinase inhibitor of angiogenic, stromal and oncogenic receptor tyrosine kinases with potent preclinical antitumor activity. Int J Cancer. 2011;129: 245–255. 10.1002/ijc.25864 21170960

[11] Fan LC, Teng HW, Shiau CW, Lin H, Hung MH, Chen YL, et al SHP-1 is a target of regorafenib in colorectal cancer. Oncotarget. 2014;5: 6243–6251. 25071018 10.18632/oncotarget.2191 PMC4171626

[12] Jung KH, Lee JH, Park JW, Paik JY, Quach CH, Lee EJ, et al Annexin V imaging detects diabetes-accelerated apoptosis and monitors the efficacy of benfotiamine treatment in ischemic limbs of mice. Mol Imaging. 2014;13: 1–7. 24824853

[13] Neves AA, Brindle KM. Imaging cell death. J Nucl Med. 2014;55: 1–4. 10.2967/jnumed.112.114264 24385310

[14] Kovar JL, Simpson MA, Schutz-Geschwender A, Olive DM. A systematic approach to the development of fluorescent contrast agents for optical imaging of mouse cancer models. Anal Biochem. 2007;367: 1–12. 17521598 10.1016/j.ab.2007.04.011

[15] Eisenblatter M, Ehrchen J, Varga G, Sunderkotter C, Heindel W, Roth J, et al In vivo optical imaging of cellular inflammatory response in granuloma formation using fluorescence-labeled macrophages. J Nucl Med. 2009;50: 1676–1682. 10.2967/jnumed.108.060707 19759121

[16] Schellenberger EA, Bogdanov A Jr., Petrovsky A, Ntziachristos V, Weissleder R, Josephson L. Optical imaging of apoptosis as a biomarker of tumor response to chemotherapy. Neoplasia. 2003;5: 187–192. 12869301 10.1016/S1476-5586(03)80050-7 PMC1502408

[17] Manning HC, Merchant NB, Foutch AC, Virostko JM, Wyatt SK, Shah C, et al Molecular imaging of therapeutic response to epidermal growth factor receptor blockade in colorectal cancer. Clin Cancer Res. 2008;14: 7413–7422. 10.1158/1078-0432.CCR-08-0239 19010858 PMC2657180

[18] Qin H, Zhang MR, Xie L, Hou Y, Hua Z, Hu M, et al PET imaging of apoptosis in tumor-bearing mice and rabbits after paclitaxel treatment with (18)F(-)Labeled recombinant human His10-annexin V. Am J Nucl Med Mol Imaging. 2015;5: 27–37. 25625024 PMC4299778

[19] Lehner S, Todica A, Vanchev Y, Uebleis C, Wang H, Herrler T, et al In vivo monitoring of parathyroid hormone treatment after myocardial infarction in mice with [68Ga]annexin A5 and [18F]fluorodeoxyglucose positron emission tomography. Mol Imaging. 2014;13. 10.2310/7290.2014.00035 25249170

[20] Lederle W, Arns S, Rix A, Gremse F, Doleschel D, Schmaljohann J, et al Failure of annexin-based apoptosis imaging in the assessment of antiangiogenic therapy effects. EJNMMI Res. 2011;1: 26 10.1186/2191-219X-1-26 22214377 PMC3251208

[21] Zhang CC, Yan Z, Giddabasappa A, Lappin PB, Painter CL, Zhang Q, et al Comparison of dynamic contrast-enhanced MR, ultrasound and optical imaging modalities to evaluate the antiangiogenic effect of PF-03084014 and sunitinib. Cancer Med. 2014;3: 462–471. 10.1002/cam4.215 24573979 PMC4101737

[22] Eisenblatter M, Holtke C, Persigehl T, Bremer C. Optical techniques for the molecular imaging of angiogenesis. Eur J Nucl Med Mol Imaging. 2010;37 Suppl 1: S127–137. 10.1007/s00259-010-1514-1 20632173

[23] Witjes JA, Babjuk M, Gontero P, Jacqmin D, Karl A, Kruck S, et al Clinical and Cost Effectiveness of Hexaminolevulinate-guided Blue-light Cystoscopy: Evidence Review and Updated Expert Recommendations. Eur Urol. 2014. 10.1016/j.eururo.2014.06.037 25001887

[24] Metildi CA, Kaushal S, Luiken GA, Hoffman RM, Bouvet M. Advantages of fluorescence-guided laparoscopic surgery of pancreatic cancer labeled with fluorescent anti-carcinoembryonic antigen antibodies in an orthotopic mouse model. J Am Coll Surg. 2014;219: 132–141. 10.1016/j.jamcollsurg.2014.02.021 24768506 PMC4065820

[25] Sheth RA, Heidari P, Esfahani SA, Wood BJ, Mahmood U. Interventional optical molecular imaging guidance during percutaneous biopsy. Radiology. 2014;271: 770–777. 10.1148/radiol.14131880 24520946 PMC4263633

[26] Behbahaninia M, Martirosyan NL, Georges J, Udovich JA, Kalani MY, Feuerstein BG, et al Intraoperative fluorescent imaging of intracranial tumors: a review. Clin Neurol Neurosurg. 2013;115: 517–528. 10.1016/j.clineuro.2013.02.019 23523009

[27] Koch M, Glatz J, Ermolayev V, de Vries EG, van Dam GM, Englmeier KH, et al Video-rate optical flow corrected intraoperative functional fluorescence imaging. J Biomed Opt. 2014;19: 046012 10.1117/1.JBO.19.4.046012 24752380

[28] Keereweer S, Van Driel PB, Robinson DJ, Lowik CW. Shifting focus in optical image-guided cancer therapy. Mol Imaging Biol. 2014;16: 1–9. 10.1007/s11307-013-0688-x 24037176

[29] Carter NJ. Regorafenib: a review of its use in previously treated patients with progressive metastatic colorectal cancer. Drugs Aging. 2014;31: 67–78. 10.1007/s40266-013-0140-6 24276917

[30] Reshef A, Shirvan A, Akselrod-Ballin A, Wall A, Ziv I. Small-molecule biomarkers for clinical PET imaging of apoptosis. J Nucl Med. 2010;51: 837–840. 10.2967/jnumed.109.063917 20484422

[31] Nguyen QD, Challapalli A, Smith G, Fortt R, Aboagye EO. Imaging apoptosis with positron emission tomography: 'bench to bedside' development of the caspase-3/7 specific radiotracer [(18)F]ICMT-11. Eur J Cancer. 2012;48: 432–440. 10.1016/j.ejca.2011.11.033 22226480

[32] Silva MT. Secondary necrosis: the natural outcome of the complete apoptotic program. FEBS Lett. 2010;584: 4491–4499. 10.1016/j.febslet.2010.10.046 20974143

[33] Krysko DV, Vanden Berghe T, D'Herde K, Vandenabeele P. Apoptosis and necrosis: detection, discrimination and phagocytosis. Methods. 2008;44: 205–221. 10.1016/j.ymeth.2007.12.001 18314051

[34] Sawai H, Domae N. Discrimination between primary necrosis and apoptosis by necrostatin-1 in Annexin V-positive/propidium iodide-negative cells. Biochem Biophys Res Commun. 2011;411: 569–573. 10.1016/j.bbrc.2011.06.186 21763280

[35] Schmieder R, Hoffmann J, Becker M, Bhargava A, Muller T, Kahmann N, et al Regorafenib (BAY 73–4506): antitumor and antimetastatic activities in preclinical models of colorectal cancer. Int J Cancer. 2014;135: 1487–1496. 10.1002/ijc.28669 24347491 PMC4277327

